# National Black HIV/AIDS Awareness Day —February 7, 2013

**Published:** 2013-02-01

February 7 is National Black HIV/AIDS Awareness Day, an observance intended to raise awareness of human immunodeficiency virus (HIV)/acquired immunodeficiency syndrome (AIDS) and encourage action to reduce the disproportionate impact of HIV/AIDS on blacks or African Americans in the United States. Compared with other races and ethnicities, blacks or African Americans had the highest HIV prevalence in 2009 (1) and the highest incidence in 2010 (2), with an estimated HIV incidence of 68.9 per 100,000 population, which was 7.9 times the rate in whites (8.7). Two of the three goals of the National HIV/AIDS Strategy are to reduce HIV incidence and HIV-related disparities (3).

In 2010, among black or African American females, heterosexual contact with a person known to have, or to be at high risk for, HIV infection was associated with an estimated 87% of new infections (2). From 2008 to 2010, the number of new infections among black or African American females decreased 21%, from 7,700 to 6,100. By comparison, the rate of new HIV infections for black or African American females (38.1 per 100,000 population) in 2010 was 20.1 times the rate for white females (1.9).

In 2010, among black or African American males in the United States, male-to-male sexual contact was associated with an estimated 72% of new HIV infections. Among black or African American men who have sex with men, males aged 13–24 years accounted for 45% of new HIV infections. This group had the highest HIV incidence of any age and racial/ethnic subgroup. The number of new HIV infections among black or African American males was stable at 14,400 in 2008 and 14,700 in 2010. By comparison, the rate of new HIV infections for black or African American males (103.6 per 100,000 population) in 2010 was 6.6 times the rate for white males (15.8).

National Black HIV/AIDS Awareness Day is an opportunity to increase HIV prevention activities, such as HIV testing, and to link persons with HIV to effective HIV medical care that reduces morbidity, mortality, and HIV transmission (4). Additional information about National Black HIV/AIDS Awareness Day is available at http://www.cdc.gov/features/blackhivaidsawareness. Additional information regarding blacks or African Americans and HIV/AIDS is available at http://www.cdc.gov/hiv.
